# Quarantine for SARS, Taiwan

**DOI:** 10.3201/eid1102.040190

**Published:** 2005-02

**Authors:** Ying-Hen Hsieh, Chwan-Chuan King, Cathy W. S. Chen, Mei-Shang Ho, Jen-Yu Lee, Feng-Chi Liu, Yi-Chun Wu, Jiunn-Shyan JulianWu

**Affiliations:** * National Chung Hsing University, Taichung, Taiwan;; † National Taiwan University, Taipei, Taiwan;; ‡ Feng Cha University, Taichung, Taiwan;; § Academia Sinica, Taipei, Taiwan;; ¶ Center for Disease Control, Taipei, Taiwan

**Keywords:** SARS, emerging infectious disease, quarantine, intervention, Taiwan, research

## Abstract

During the 2003 outbreak of severe acute respiratory syndrome (SARS) in Taiwan, >150,000 persons were quarantined, 24 of whom were later found to have laboratory-confirmed SARS-coronavirus (SARS-CoV) infection. Since no evidence exists that SARS-CoV is infective before the onset of symptoms and the quarantined persons were exposed but not symptomatic, we thought the quarantine's effectiveness should be investigated. Using the Taiwan quarantine data, we found that the onset-to-diagnosis time of previously quarantined confirmed case-patients was significantly shortened compared to that for those who had not been quarantined. Thus, quarantine for SARS in Taiwan screened potentially infective persons for swift diagnosis and hospitalization after onset, thereby indirectly reducing infections. Full-scale quarantine measures implemented on April 28 led to a significant improvement in onset-to-diagnosis time of all SARS patients, regardless of previous quarantine status. We discuss the temporal effects of quarantine measures and other interventions on detection and isolation as well as the potential usefulness of quarantine in faster identification of persons with SARS and in improving isolation measures.

## Abstract 2

Quarantine for SARS during the 2003 Taiwan outbreak expedited case detection, thereby indirectly preventing infections.

The severe acute respiratory syndrome (SARS) epidemic from November 2002 to June 2003 came with much public attention and left swiftly, resulting in >8,000 probable cases worldwide and 774 deaths (*1*). Prominent among retrospective analyses is the belief that the simple ancient system of placing persons suspected of being infected under quarantine was instrumental in the quick containment of the outbreak (*2**–**5*). However, questions persist regarding how quarantine worked to control this disease, given the time-tested axiom that quarantine is most useful only when patients are infectious before becoming symptomatic, thus directly preventing secondary infections (*6*). Moreover, due to early confusion resulting from imprecise clinical diagnosis and case definition (*7*), correct clinical diagnosis and prompt isolation were often impossible, which resulted in insufficient isolation and gaps in the containment strategy for hospital infection control (*8*). Since all available evidence indicates that SARS patients were only infectious after symptom onset (*9*), one may argue that quarantine provides a window of several days during which illnesses can be diagnosed swiftly and persons isolated accordingly. In this study, we used data from the Taiwan SARS outbreak to explore whether quarantine was effective in expediting the time from onset to clinical diagnosis and hospitalization, and the time from clinical diagnosis to classification as a probable case-patient, thus contributing indirectly to prevention of possible infections.

## Methods

### Data

During the outbreak of 2003, 346 SARS cases were officially confirmed in Taiwan, among which were 37 direct SARS deaths (cause of death was recorded as SARS) and 36 SARS-related deaths (cause of death was not directly attributed to SARS) as reported by the World Health Organization (WHO) (*1*). To guard against the potential threat of a large-scale epidemic, the government attempted to place >150,000 people under home quarantine. Two distinct levels of quarantine were implemented in Taiwan. Level A quarantine, aimed at people having close contact with a suspected SARS case-patient, was implemented on March 18, 2003. Level B quarantine, aimed at travelers from affected areas, was implemented on April 28, in the aftermath of the first SARS death on April 26 (*10**,**11*). Most of the quarantined persons were confined to their homes for 10–14 days. Public health nurses would bring the quarantined persons 3 meals every day and sometimes helped them with odd jobs such as washing clothes or taking care of pets. Center for Disease Control–Taiwan officially confirmed 346 SARS-CoV–positive cases, of which 17 case-patients had been previously quarantined; 134 additional laboratory-confirmed, antibody-positive SARS cases occurred, of which 7 case-patients had previously been quarantined. The total number of confirmed SARS case-patients in Taiwan by the end of December 2004 was 480, of which 24 had been quarantined previously. Details regarding the persons quarantined during the SARS outbreak are itemized in Table 1.

**Table 1:** Cumulative numbers of persons under quarantine during the SARS outbreak, Taiwan, 2003, and the quarantined SARS patients classified by their status*

Level and reason for quarantine	No. quarantined persons	No. quarantined officially confirmed SARS-CoV case-patients	No. quarantined laboratory-confirmed, antibody-positive SARS case-patients
Level A			
Family members	7,921	8	2
Classmates and teachers	16,564	1	0
Healthcare workers	2,409	0	3
Others†	19,224	6‡	1
All others§	9,514	2	1
Subtotal	55,632	17	7
Level B	95,828	0	0
Total	151,460	17	7

*Updated December 2004. †Passengers and drivers of domestic public transportation traveling for >1 hour in the same bus or train cabin with a SARS case-patient, persons who had contacts with someone under quarantine or receiving care in a medical facility where cluster infection had occurred, and homeless persons. ‡Co-workers and friends of SARS case-patients, airplane passengers who sat within 3 rows of or stayed in the same room as SARS patients, and persons with missing information. §One case-patient had onset of symptoms 2 days after the end of quarantine.

The 134 patients with laboratory-confirmed SARS either had milder symptoms and SARS was therefore clinically diagnosed as suspected cases, ruled out at the time of the outbreak, or considered probable in patients whose specimens had previously tested negative by polymerase chain reaction (PCR) or anti–SARS-CoV antibody, perhaps due to wrong timing, but later were reconfirmed by >2 different laboratory tests in a follow-up epidemiologic study. Seven people in this group had been previously quarantined. Our criterion for a quarantined person was someone who had been placed under official quarantine for >1 day before the onset of symptoms. Thus, persons in whom symptoms developed on the same date or before the notification of quarantine were considered not quarantined and were therefore excluded. Persons who were known to have had a record of close contacts with others during the supposed quarantine period were also excluded. One of the 24 case-patients actually had an imported case but was quarantined before implementation of level B quarantine on April 28 for reasons other than simply being a traveler from an affected area.

### Statistical Analyses

We compared the mean time from onset of symptoms to clinical diagnosis (and admission) for the 24 patients with laboratory-confirmed SARS-CoV who had been quarantined before symptom onset to that of the 451 SARS-CoV–positive case-patients who had not. Note that 5 cases were deleted from the data of 480 total cases for our comparison test because of missing information on their relevant dates. (We will use the term "diagnosis" to mean clinical diagnosis hereafter). For the mean time from diagnosis to classification as probable case, we only used the officially confirmed cases for comparison, since the laboratory-confirmed cases were either ruled out or classified as suspected cases only and thus had no classification of probable time. Again, 2 of these cases were deleted from the data for our comparison test due to missing information on their relevant dates; therefore, 344 case-patients (17 quarantined and 327 nonquarantined) were used. Due to the skewed data, we used the nonparametric Mann-Whitney test.

To investigate the effect of large-scale quarantine on the changes in the efficiency of the public health system to identify SARS patients for isolation, we considered the temporal effect of important events for intervention and control of SARS in Taiwan. On April 28, level B quarantine was implemented, which marked the start of large-scale home quarantine (*12*).

A second important date in SARS prevention and control was May 10, when changes in the review and classification procedures were implemented by the cabinet-level SARS Prevention and Extrication Committee in Taiwan to expedite the review and classification of SARS cases (*13*). Before May 9, the relevant medical records (including any available laboratory test results) of all reported SARS patients were reviewed by a central SARS Advisory Committee of the Center for Disease Control–Taiwan in Taipei. Due to the rapid increase in the number of reported cases caused by the hospital cluster outbreaks in Taipei in late April, the SARS Advisory Committee in CDC-Taiwan could not handle the rapidly increasing caseload. Consequently, after May 10, 3 regional offices of the Bureau of National Health Insurance in northern, central, and southern Taiwan took over the responsibility of case review. Local SARS expert committees were established in the 3 regions with each committee consisting of specialists similar to the central committee in Taipei. The experiences and the standard operation procedures of case review and case classification used by the central committee were transferred to the 3 regional SARS expert committees in the Bureau of National Health Insurance through several consensus meetings (*14*).

We used the Mann-Whitney test to compare the time intervals from onset to clinical diagnosis of SARS symptoms of confirmed SARS-CoV case-patients with onset occurring during the 3 periods of February 25–April 27 (period 1), April 28–May 9 (period 2), and May 10–June 15 (period 3). Five patients were deleted from the data for our comparison test due to missing information on their relevant dates, and 2 patients were deleted because their onsets of SARS did not occur during the 3 time periods.

We also compared, using the Mann-Whitney test, the intervals from diagnosis to classification as probable SARS of the 343 officially confirmed SARS-CoV case-patients (by dividing the cases into 3 groups, according to the time period in which the date of diagnosis occurred). Again, the laboratory-confirmed cases had never been classified as probable cases. Moreover, 2 cases were deleted from the data for our comparison test due to missing information on their relevant dates, and 1 case was deleted because classification as a probable case-patient did not occur during the 3 periods from February 25 to June 15.

## Results

The mean time from onset to diagnosis for the previously quarantined persons (1.20 days) was significantly shorter than that of those who were not quarantined (2.89 days) (Table 2). However, the respective mean times from diagnosis to classification (6.21 days and 7.34 days) (Table 2), though slightly reduced for the quarantined persons, were not significantly different. For the mean onset-to-diagnosis time, period 1 was significantly longer (3.60 days to 2.49 days) than period 2 (p < 0.0001), while the mean difference before and after May 10 was not significant (p = 0.0722) (Table 3). The mean diagnosis-to-classification time (Table 4) was not significantly different from period 1 to period 2. However, the time was significantly shortened after May 10 (from period 2 to period 3).

**Table 2:** Comparison of mean time intervals by using the Mann-Whitney test for the onset-to-diagnosis and diagnosis-to-classification times for quarantined and nonquarantined SARS patients, Taiwan, 2003

	Onset-to-diagnosis interval (d)	Diagnosis- to-classification interval (d)
Quarantined persons	1.203 (n = 24)	7.7647 (n = 17)
Nonquarantined persons	2.8914 (n = 451)*	7.5443 (n = 327)†
Mean difference	1.6831‡ (0.0061)	0.2204 (0.7864)

*5 cases deleted because of missing information on the relevant dates. †2 cases deleted because of missing information on the relevant dates. ‡Denotes significance at the 1% level; p values of Mann-Whitney test are in parentheses.

**Table 3:** Results of Mann-Whitney test for temporal changes in onset-to-diagnosis time of 473* confirmed SARS case-patients with onset of illness during period 1,† period 2, and period 3, 2003

Interval (d)	Period 1 (N = 161)	Period 2 (N = 146)	Period 3 (N = 166)	Mean difference (p value)
Onset to diagnosis	3.6398	2.0959	–	1.5439‡ (< 0.0001)
Onset to diagnosis	–	2.0959	2.6024	0.5065 (0.0722)

*5 cases deleted because of missing information on the relevant dates and 2 case-patients deleted because their onsets of SARS did not occur during the 3 periods of 2/25–6/15. †Period 1 (2/25–4/27), time from onset of first case to the day before implementation of intervention measures including level B quarantine; period 2 (4/28–5/9), time from implementation of intervention measures to the implementation of expedited classification procedure; period 3 (5/10–6/15), time of the expedited classification procedure to the date of onset of the last SARS case. ‡Denotes significance at 1% level.

**Table 4:** Mann-Whitney test results for temporal changes in the diagnosis-to-classification time of 343* officially confirmed SARS-CoV cases with classification during period 1,† period 2, and period 3, Taiwan, 2003

Interval (d)	Period 1 (N = 103)	Period 2 (N = 114)	Period 3 (N = 126)	Mean difference (p value)
Diagnosis to classification	9.1845	8.2368	–	0.9477 (0.7729)
Diagnosis to classification	–	8.2368	5.6508	2.5860‡ (< 0.0001)

*2 cases were deleted from the data for our comparison test because of missing information on the relevant dates, and 1 case was deleted because classification as probable case did not occur during the 3 time periods of 2/25–6/15. †Period 1, time from onset of first case to the day before implementation of intervention measures including Level B quarantine; period 2, time from implementation of intervention measures to the implementation of expedited classification procedure; period 3, time of the expedited classification procedure to the date of onset of the last SARS case. ‡Denotes significance at the 1% level.

## Discussion and Conclusions

The experience in the affected areas has shown that the transmission of SARS can be prevented by adherence to basic public health measures, including rapid case detection, isolation of patients with suspected and probable cases, contact tracing, and good infection control (*9*). The effect of possible delays in effective isolation of probable case-patients has been studied in some modeling work on SARS (*15**–**17*). In Taiwan, all patients were supposed to be placed in the isolation room and negative pressure room, if available, as soon as they were reported as having probable or suspected SARS. For most of May, the number of suspected case-patients alone remained well above 1,000, partly because of confusion in diagnosis and the tendency to overdiagnosis because of heightened alertness on the part of physicians and legal punishment for underreporting. At times, however, due to the lack of available isolation rooms or the number of suspected cases pending review, patients with suspected but unconfirmed SARS were kept for days in an observation room or emergency department under crude isolation, where nosocomial infections readily occurred. At other times, patients scheduled to transfer to another hospital with negative pressure isolation rooms were temporarily kept in the observation room in the emergency department where nosocomial infections might occur because of insufficient isolation and protection procedures (*18*). When full isolation facilities were not available to all patients, those classified as probable SARS case-patients likely received higher priority and were observed more closely during their isolation by healthcare workers than were the suspected case-patients.

For some case-patients, delays occurred because of the patient's uncertain status or urgent need for intubation without comprehensive information on the patient's contact and clinical history; these delays led to insufficient protection and isolation. One well-known case-patient was the index patient at Hoping Hospital in Taipei, where the largest cluster infection in Taiwan occurred. Her condition was diagnosed and reported as suspected SARS on April 9. However, because the patient had no apparent contact with another known SARS case-patient, her case was reviewed but not reclassified as probable until April 25, by which time the clustered cases, which included medical staff members and an x-ray technician who had contact with her, had already forced the hospital to shut down on the previous day. More strict infection control would have been in place had the index patient been confirmed as a probable SARS patient. Several other similar cases occurred in Taiwan, some more than 1 month later. Therefore the importance of rapid classification as probable case-patients cannot be ignored.

Our results show that quarantine reduced the time from onset to diagnosis but did not significantly reduce the time from diagnosis to classification. Thus, a previously quarantined person could expect his or her condition to be diagnosed and to be hospitalized more quickly once clinical symptoms appeared. However the same person would not receive higher priority in the classification process to determine candidates for effective isolation. Nevertheless, in many hospitals with available isolation rooms, patients with suspected cases were effectively isolated as soon as chest radiographic evidence of infiltrates consistent with pneumonia or acute respiratory distress syndrome became available. Moreover, in the latter stages of the epidemic when a reliable laboratory test for SARS-CoV became more available, many patients were isolated in negative pressure chambers immediately if results of reverse transcription–PCR for SARS-CoV from 2 different laboratories were positive. Therefore, the effect of classification as a probable case-patient might not be as pronounced as it would have been otherwise.

For all laboratory-confirmed case-patients, regardless of whether they were quarantined previously, the implementation of full-scale intervention measures, including level B quarantine on April 28, significantly decreased the time from onset to diagnosis, but it only slightly improved the time from diagnosis to classification. However, the small sample size of 24 previously quarantined SARS case-patients did not permit a meaningful test of whether a significant difference existed for the previously quarantined persons during each of the 3 periods.

By comparison, the change in the review and classification procedure initiated on May 10 helped shorten the diagnosis-to-classification time for all SARS patients, indicating that the action by the SARS Prevention and Extrication Committee to expedite the review process had indeed worked. However, by separating the analyses of data into discrete epochs marked by significant events, we have included those cases whose illnesses straddle epochs.

In the future, when facing newly emerging infectious diseases like SARS, in which the patient's infectivity in the incubation period is unknown, precise clinical diagnosis cannot be made, and modes of transmission are uncertain, quarantine should be used not only to directly prevent possible asymptomatic infections but also to screen out potentially infective persons to swiftly prevent secondary or even tertiary infections after the symptoms appear.

The quarantine in Taiwan was indeed useful in helping to identify persons who are likely to develop symptoms and isolate them more quickly if and when they did, although its effect on isolation and infection control could perhaps be improved by quicker classification or confirmation of previously quarantined patients. No conclusion was drawn regarding whether better outbreak control would be achieved by placing fewer persons in quarantine or by concentrating on improving the efficiency of detection and isolation procedures. In fact, each area may be improved in efficiency without jeopardizing the other's improvement.
